# *GBA2* Mutations Cause a Marinesco-Sjögren-Like Syndrome: Genetic and Biochemical Studies

**DOI:** 10.1371/journal.pone.0169309

**Published:** 2017-01-04

**Authors:** Kristoffer Haugarvoll, Stefan Johansson, Carlos E. Rodriguez, Helge Boman, Bjørn Ivar Haukanes, Ove Bruland, Francisco Roque, Inge Jonassen, Maria Blomqvist, Wenche Telstad, Jan-Eric Månsson, Per Morten Knappskog, Laurence A. Bindoff

**Affiliations:** 1 Department of Neurology, Haukeland University Hospital, Bergen, Norway; 2 Department of Clinical Medicine (K1), University of Bergen, Bergen, Norway; 3 Center for Medical Genetics and Molecular Medicine, Haukeland University Hospital, Bergen, Norway; 4 Department of Clinical Science, University of Bergen, Bergen, Norway; 5 Department of Clinical Chemistry and Transfusion Medicine, Institute of Biomedicine, Sahlgrenska Academy, University of Gothenburg, Gothenburg, Sweden; 6 Computational Biology Unit, Department of Informatics, University of Bergen, Bergen, Norway; 7 Department of Neurology, Førde Hospital, Førde, Norway; Azienda Ospedaliero-Universitaria Santa Maria della Misericordia, ITALY

## Abstract

**Background:**

With the advent new sequencing technologies, we now have the tools to understand the phenotypic diversity and the common occurrence of phenocopies. We used these techniques to investigate two Norwegian families with an autosomal recessive cerebellar ataxia with cataracts and mental retardation.

**Methods and Results:**

Single nucleotide polymorphism (SNP) chip analysis followed by Exome sequencing identified a 2 bp homozygous deletion in *GBA2* in both families, c.1528_1529del [p.Met510Valfs*17]. Furthermore, we report the biochemical characterization of GBA2 in these patients. Our studies show that a reduced activity of GBA2 is sufficient to elevate the levels of glucosylceramide to similar levels as seen in Gaucher disease. Furthermore, leucocytes seem to be the proper enzyme source for in vitro analysis of GBA2 activity.

**Conclusions:**

We report *GBA2* mutations causing a Marinesco-Sjögren-like syndrome in two Norwegian families. One of the families was originally diagnosed with Marinesco-Sjögren syndrome based on an autosomal recessive cerebellar ataxia with cataracts and mental retardation. Our findings highlight the phenotypic variability associated with *GBA2* mutations, and suggest that patients with Marinesco-Sjögren-like syndromes should be tested for mutations in this gene.

## Introduction

Mutations in the non-lysosomal glucosylceramidase 2 gene *(GBA2; ENSG00000070610)* have been identified as a novel cause of autosomal recessive ataxia and lower extremity spasticity by two groups independently [1,2]. The clinical syndrome associated with *GBA2* mutations was characterized either as an autosomal recessive cerebellar ataxia (ARCA) with spasticity or complicated hereditary spastic paraplegia (HSP) with ataxia: the latter is also known as spastic paraplegia 46 [*SPG46* (MIM #614409)] [2,3]. Additional features include cataracts, peripheral neuropathy, skeletal deformities, mild to moderate mental impairment and hearing loss [1,2,4,5].

Marinesco-Sjögren syndrome [MSS (MIM 248800)] is an autosomal recessive disorder characterized by cerebellar atrophy with ataxia, early-onset cataracts, hypotonia and muscle weakness. Additional features include mild to severe intellectual disability, short stature and various skeletal abnormalities including scoliosis [6–9]. Children with Marinesco-Sjögren syndrome usually present with muscular hypotonia in early infancy; distal and proximal muscular weakness develops during the first decade and subsequently, cerebellar ataxia, and dysarthria become apparent. Motor function worsens progressively, and then stabilizes at an unpredictable age and degree of severity. Cataracts can develop rapidly and typically require lens extraction in the first decade of life. Although many adults are severely handicapped, life span in MSS appears to be near normal. Autosomal recessive mutations in the SIL1 nucleotide exchange factor (*SIL1; ENSG00000120725*) gene are the only known cause of MSS [10,11]. *SIL1* mutations are identified in about 50–60% of patients with the characteristic MSS triad (myopathy, early-onset cataracts, and cerebellar ataxia) [12].

**Fig 1:**
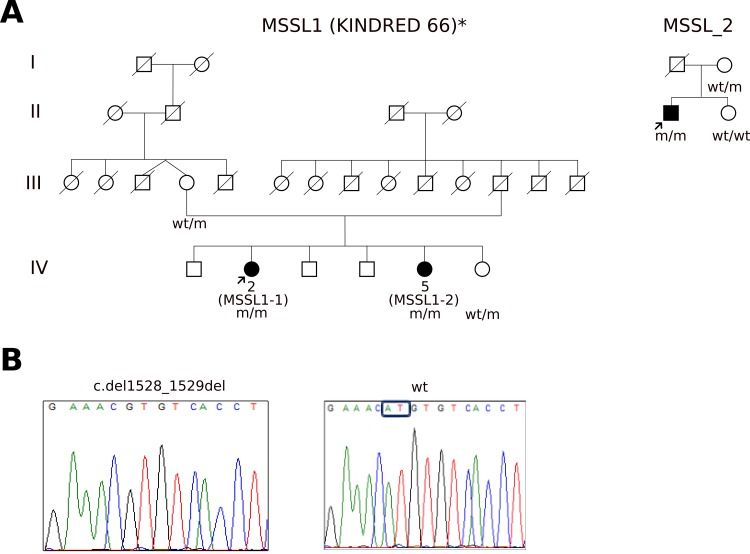
Pedigrees with homozygous c.1528_1529del [p.Met510Valfs*17] *GBA2* mutation A) Kindred 66 was originally reported by Skre and Berg [13]. Arrow: probands; Squares: males; circles: females; diagonal line: deceased individual; Black symbols: affected individuals; white symbols: unaffected individuals; w/w: homozygous for the wild-type allele; w/m: heterozygous for the *GBA2* c.1528_1529del mutation; m/m: homozygous for the *GBA2* c.1528_1529del mutation. B) Sequencing of the *GBA2* transcript. Sequencing of the GBA2 transcript using RNA purified from cultured patient fibroblasts showed homozygosity for the 2 bp deletion (c.1528_1529del).

We report the identification of a novel homozygous *GBA2* mutation by exome sequencing in two Norwegian families with a Marinesco-Sjögren-like (MSSL) syndrome without *SIL1* mutations (Fig 1). One family was originally reported as having MSS by Skre and Berg (Kindred 66) in 1977 [13]. The same novel *GBA2* mutation was identified in an additional, apparently unrelated patient. In addition, we also report the biochemical characterization of GBA2 (Q9HCG7) activity in patients using a method developed for measuring GBA2 activity in leukocytes.

## Methods

### Standard protocol approvals, registrations, and patient consents

Our study was approved by the Regional Committee for Medical and Health Research Ethics, Western Norway (IRB00001872). All study participants provided written informed consent.

### Patients

Two affected sisters (MSSL1-1, MSSL1-2) who belonged to a family from western Norway previously reported as Kindred 66 by Skre and Berg [13]. A third unrelated patient MSSL2-1 with a similar phenotype was included in this study. The patients underwent repeated neurological examinations and their medical records were examined as a part of this study.

### Homozygozity mapping and SIL1 mutation screening

DNA was purified from EDTA blood using the QiaSymphony system (Qiagen, Hilden, Germany). Genome-wide single nucleotide polymorphism (SNP) genotyping was performed with the Genome Wide Human SNP array 50K (Affymetrix, Santa Clara, USA) and analysed using PLINK v1.07 [14]. For homozygozity mapping, we searched for any region >3 Mb, with minimum of 30 SNPs and less than four heterozygous calls. Recessive *SIL1* mutations were excluded by haplotype analysis and direct sequencing of the *SIL1* gene.

### Whole-exome sequencing

Whole exome sequencing was performed at HudsonAlpha Institute for Biotechnology (Huntsville, AL, USA) using Roche-NimbleGen Sequence Capture EZ Exome v2 kit and paired-end 100nt sequencing on the Illumina HiSeq [15]. The 8.9 Giga-bases of aligned sequence data resulted in 100X median coverage of the target capture regions with more than 97% of target bases covered at least 8X. Screening of candidate mutations in further family members, patients and control individuals was done by Sanger sequencing.

### RNA analysis

Skin biopsies were taken from the forearm and cultured in Amniochrome II Basal medium with Amniochrome II Modified Supplement (Lonza, Bazel, Switzerland) at 37oC in 5% CO2. The human lens epithelial cell line HLE-B3 (ATCC, CRL-11421, Manassas, VA, USA) was grown in Minimum Essential Medium Eagle (Sigma-Aldrich, St. Louis, MO, USA) supplemented with Fetal Bovine Serum, L-glutamine and 1x penicillin-streptomycin (Sigma-Aldrich) at 37oC in 5% CO2.

Total RNA was isolated from fibroblasts obtained from patients and controls by using the RNeasy Mini Kit (QIAGEN, Hilden, Germany) for fibroblasts The concentration and quality of RNA was determined by using Nanodrop ND-1000 (Thermo Fisher Scientific, Waltman, MA, USA) and the Experion Automated Electrophoresis System with RNA StdSens Chips (BioRad, Hercules, CA, USA). cDNA was synthesized by reverse transcriptase PCR using the SuperScript VILO cDNA synthesis kit including SuperScript III reverse transcriptase (Thermo Fisher Scientific) as described by the manufacturer.

The region harboring the 2 bp deletion and the adjacent exons were amplified by PCR from the patient derived´s cDNA and the PCR products were sequenced by the BigDyeterminator ver 1.1 kit (Applied Biosystems) and analysed on 3730/3730xl DNA Analyzer from Applied Biosystems and further analyzed using the Sequencing Analysis software v. 6.0 (Thermo Fisher Scientific) (Sequence Scanner Version 1.0).

### Measurement of glucosylceramide:

Glucosylceramide concentration was determined in plasma and erythrocytes using our routine procedure for monitoring the efficacy of enzyme replacement therapy (ERT) in Gaucher patients (MIM #230800) [16]. Lipids extracts from plasma or erythrocytes were purified by silica gel column chromatography and the glucosylceramide fraction eluted with chloroform-methanol 85:15 (v/v). The glucosylceramide concentrations were determined with high-performance thin-layer chromatography (HPTLC) followed by orcinol detection and densitometric evaluation [17,18].

### Enzymatic activity measurement of GBA2

Leukocytes were isolated from ~6 mL EDTA blood as previously described [19], and kept at -20°C for 1 day before being thawed and maintained on ice until the start of the enzyme reaction. Leukocytes were diluted in 300 μL of deionized water and homogenized at 4°C (glass/Teflon; 10 strokes). The homogenate was sonicated (ultrasonic bath Branson 3800, 40 kHz, Emerson industrial Automation, St Louis, MO, USA) in a glass tube for 1 minute, rested 30 seconds on ice and sonicated for an additional minute. Disrupted cells were used to measure enzyme activity within 3 hours. Protein concentration was determined (BCA Protein Assay Reagent method, Pierce, Rockford, USA) and 10 to 40 μg/sample was suitable to accomplish the analysis.

Stock solutions of substrate and inhibitor were prepared as follows: Substrate: 4-metylumbelliferon-β-glucopyranoside 7.4 mM (Koch-Light, Haverhill, Suffolk, UK) in citric acid 80 mM (Sigma-Aldrich, St. Louis, MO, USA) and disodium hydrogen phosphate 240 mM (Merck, VWR International), pH 5.8. Inhibitor: AMP-DNM: AMP-Deoxynojirimycin 42 nM (Carbosynth, Compton, Berkshire, UK). The product, presented in ethanol solution, was diluted with deionized water with no presence of turbidity. Fifteen– 40 μl of sample (~10–40 μg protein) with and without 10 μL of inhibitor were made up to 50 μl with deionized water and pre-incubated for 1 min at 37°C. The assay was initiated by adding 50 μl of substrate. Duplicates of each sample with and without inhibitor, as well as quadruplicate blanks (50 μl of deionized water and 50 μl of substrate) were incubated in 1.5 mL eppendorf tubes for 30 minutes at 37°C in a water bath with orbital shaker. The reaction was stopped with 0.6 mL glycine 0.25 M, pH 10.3 (Merck) and fluorescence of samples, blanks and standard solution (4-Methyl Umbelliferon 1 μM, diluted in Glycine 0.25M, pH 10.3) measured within 1 hour (shielded from light) in a spectrofluorometer (Jasco FP-6500, Jasco Inc, Easton, MD, USA) using an excitation wavelength of 360 nm and an emission wavelength of 448 nm. The activity of GBA2 was calculated according to the following equations and expressed as μkatal/kg protein.

Sample fluorescence-Substrate blank fluorescence*7000001800*standard fluorescence=fkataltest

fkataltestμgproteintest=μkatalkgprotein

Where:

$$\mathrm{Std conc}\times \mathrm{Sample vol}=10^{-6}\mathrm{mol}/L\times 0,710^{-3}L\times 10^{15}\mathrm{fmol}/\mathrm{mol}=700000\;\mathrm{fmol}$$

$$\mathrm{Reaction time}\left(s\right)=1800$$

GBA2 activity=Activity without inhibitor-Activity with inhibitor

## Results

### Clinical features

The clinical features are summarized in the Table 1. All three patients developed normally initially. Disease onset was between 5–7 years and all developed a clinical syndrome consisting of cerebellar ataxia, lower limb spasticity, urge incontinence, intellectual disability, peripheral neuropathy and bilateral cataracts. Impaired vision due to bilateral cataracts was the initial symptom in Patient MSSL1-2 (Table 1). The two other patients developed cataracts later; in patient MSSL1-1 cataracts were noted in her 30s and she underwent bilateral cataract surgery at age 49 years. The proband from family MSSL2 developed bilateral cataracts in early adulthood and underwent bilateral cataract surgery at age 26 years.

**Table 1:** Clinical features of patients harbouring the homozygous c.1528_1529del [p.Met510Valfs*17] GBA2 mutation.

	MSSL1-1(Kindred 66 IV:2 [13])	MSSL1-2(Kindred 66: IV:5 [13])	MSSL2-1
Age at last examination	70 yrs.	48 yrs.	51 yrs.
Age of onset	5 yrs.	6 yrs.	7 yrs.
First symptom	Poor balance with unsteady gait	Visual impairment	Poor balance with unsteady gait
Cataract	+	+	+
Ataxia	+	+	+
Dysarthria	+	+	+
Spasticity	+/10	+	+
Intellectual disability	+	+	+
Autonomic dysfunction	Urge incontinence	Urge incontinence	Urge incontinence
Tremor / abnormal movements	P&I tremor in UL and head tremor	P&I tremor in upper limbs and head. Later athetosis with retroversion & abn. neck movements	-
Scoliosis	-	+	-
Pes cavus	-	-	+
MRI	NA	Generalized cerebral and cerebellar atrophy with thinning of the corpus callosum	Generalized cerebral and cerebellar atrophy with thinning of the corpus callosum
ENMG	Axonal degeneration.	Distal amyotrophy	Axonal-demyelinating sensorimotor neuropathy
Muscle biopsy	Neurogenic atrophy	NA	Neurogenic atrophy
Glucosylceramide μmol/L			
Erythrocytes control range: 1.8–6.0	15.7	20.1	23.8
Plasma control range: 4.7–9.7	29.0	37.4	27.2

EMNG: electroneuromyography; MRI: magnetic resonance imaging; NA: not available; P&I: positional and intentional; UL: upper limbs; yrs: years; +: present; -: absent.

Muscle biopsy performed in MSSL1-1 and MSSL2-1 revealed findings consistent with neurogenic atrophy comprising groups of atrophic fibres, hypertrophied fibres and increased numbers of internal nuclei. Two of the three patients had skeletal deformities comprising pes cavus or scoliosis. The clinical course was slowly progressive and at the last examination the clinical picture was dominated by severe spasticity with hyperreflexia and plantar inversion in all limbs with severe cerebellar ataxia, urinary incontinence, and severe dysarthria. At this point, all were wheelchair-bound and required physical assistance.

### Molecular findings

Information obtained from church records revealed several possible consanguineous connections between the MSSL1 parents and the mother of MSSL2-1. We, therefore, searched for regions of shared homozygosity: the two sisters (MSSL1-1, 1–2) shared one large region of homozygosity on chr 9: 27,550,594–36,866,740 (build 36.3, ~7.8 cM) and a subset of the region appeared to be shared by MSSL2-1 (Chr 9:33,019,468–36,866,740; ~2.8 cM). This region contained 120 genes, none of which had been linked to MSS-like phenotypes at that time.

We performed whole exome sequencing of the proband in both families. Variants were filtered against 1000Genomes data and our in-house variant frequency database (minor allele frequency; MAF< 0.5) to 288 and 303 variants respectively in each patient. Using the pre-existing homozygosity data to limit our search, we identified a homozygous 2 bp deletion in *GBA2* in both probands. The mutation, c.1528_1529del, is located in exon 9 and predicted to introduce a frameshift and premature stop codon p.Met510Valfs*17. The mutation was confirmed by Sanger sequencing to be homozygous in all three affected, heterozygous in the parents (Fig 1) and absent in 192 healthy blood donors and more than 500 in house exomes. No variants were identified in the *SIL1* gene. Fibroblasts from MSSL1-2 were cultured and RTPCR showed the expression of a transcript containing 2 bp deletion identified by exome sequencing.

### Biochemical findings

All patients had significantly increased concentrations of glucosylceramide both in erythrocytes and plasma and values were higher than in many non-treated Gaucher patients (Table 1). The activity of glucosylceramidase in lymphocytes and chitotriosidase in plasma was normal in patient MSSL2-1 (data not shown) and therefore, a diagnosis of Gaucher was not investigated further in the patients MSSL1-1 and MSSL1-2.

Enzymatic determination of the GBA2 activity in leukocytes showed reduced activity of GBA2 in MSSL1-2 (0.2 μkatal/kg protein) corresponding to a residual activity of 7% compared with the mean value of 15 controls (2.7; reference range: 1.2–4.3 μkatal/kg protein).

## Discussion

We report the clinical features and genetic diagnosis in two families from western Norway who were originally diagnosed with Marinesco-Sjøgren syndrome based on the presence of autosomal recessive cerebellar ataxia, congenital cataract and mental retardation [13]. *SIL1* mutations were excluded by haplotype analysis and direct sequencing. Exome sequencing identified a novel homozygous c.1528_1529del [p.Met510Valfs*17] *GBA2* mutation as the cause of the phenotype in both families (Fig 1). This is a very rare variant, reported with an allele frequency of 8.7 x 10^−5^ (Latino population) and 4.5x 10^−5^ (European population) in the Exome Aggregation Consortium (ExAC) database (http://exac.broadinstitute.org/). This deletion is present in the mRNA and is predicted to be translated into a protein lacking > 400 C-terminal aminoacids. Further, we developed an enzymatic assay for GBA2, which confirmed the profound loss of activity.

It has been shown that recessive *SIL1* mutations are found in ~60% of patients with the clinical Marinesco-Sjögren syndrome triad (myopathy, early-onset cataracts, and cerebellar ataxia) [12]. Muscle biopsy from patients with *SIL1*-mutations shows various non-specific features that indicate skeletal muscle fibre degeneration. At the ultrastructural level, degenerating myonuclei are occasionally surrounded by an electron-dense, membrane-like structure that may be characteristic of pathology associated *SIL1* mutations [12,20,21]. In contrast, biopsy changes in our patients were more consistent with amyotrophy, i.e. neurogenic atrophy (Table 1). Furthermore, CK was normal in all patients.

In most, but not all, cases of *SIL1*-associated Marinesco-Sjögren syndrome, cataracts manifest before the age of seven and are bilateral. While all of our patients developed cataract, only one of them did so before the age of seven, but all underwent removal of cataract at some stage. Mental retardation varied from mild to moderate, and both sisters have gone from requiring help with daily activities to complete dependency in the last 10 years. Our patients all developed distal amyotrophy i.e. neurogenic wasting and, in two, electrophysiological studies showed axonal loss with one having additional demyelinating changes.

*GBA2* mutations have been described in families manifesting complex early onset recessive phenotypes usually involving spasticity, but also ataxia and peripheral nerve involvement. Cataract is reported, but not in all families [1], and in those reported to have this feature it was not made clear whether they were early onset [2]. Two of the reported cases manifested testicular hypotrophy and abnormalities of spermatozoa [2]. Gonadal dysfunction was also reported in two of our patients (MSSL1—the sisters from Kindred 66) although this was not treated or defined further after the initial report [13]. Imaging findings range from normal MRI to cerebellar atrophy and thinning of the corpus callosum similar to that seen in 2/3 of our patients [1,2].

Alkylated deoxynojirimycins are extremely potent inhibitors of GBA2 [22] and the drug Zavesca® (Miglustat, *N*-butyl-deoxynojirimycin) is used as a substrate reduction therapy (for GBA1) in type 1 Gaucher patients. Following initiation of Miglustat therapy, Gaucher patients show even higher concentrations of glucosylceramide in erythrocytes than before therapy (J-E Månsson, unpublished results). Similarly, in all three patients included in this study, the concentration of glucosylceramide in plasma was similar to that observed in untreated Gaucher patients while in erythrocytes, the concentration was higher [16]. Further, in an experimental study of the Niemann-Pick C mice model, Miglustat was also found to increase the glucosylceramide concentration in the brain [23], and has been shown to stabilize neurological symptoms in humans [24]. Results from different studies suggest that GBA2 might be a modifying factor in the phenotypic expression of at least some Gaucher disease patients [25], however, we show that a defect in GBA2 is sufficient to elevate the levels of glucosylceramide to similar levels as seen in patients with Gaucher disease.

Having identified the genetic defect and the raised glucosylceramide concentration, we developed an assay to measure specific GBA2 activity using the synthetic substance 4-methylumbelliferyl-beta-D-glucopyranoside (4-MU-beta-glc) as substrate. Since this substance has affinity for two additional enzymes, GBA1 (lysosomal glucosylceramidase) and GBA3 (cytosolic glucosylceramidase), and specific inhibitors such as Conduritol B epoxide (CBE) for GBA1 [22] do not inhibit GBA3 [26], we chose the compound AMP-deoxynojirimycin that strongly inhibits GBA2, but not to the same extent GBA1 nor GBA3 [27] to measure GBA 2 activity by subtraction. The importance of exploring the possible inhibition by AMP-deoxynojirimycin of both GBA1 and GBA3 is supported by the findings of variable expression of cytosolic beta-glucosidase and glucocerebrosidase activity in leucocytes [28]. We also tested fibroblasts as an enzyme source, but were not able to detect any GBA2 activity in these cells. Thus, leucocytes seem to be the proper enzyme source for *in vitro* analysis of GBA2.

The degree of clinical variation shown by families with GBA2 mutations is not unusual, but means that we must be vigilant when confronted by complex phenotypes. Two of our patients were initially given a diagnosis of Marinesco-Sjögren syndrome and while this was based on clinical understanding at that time, there are certain to be other families with complicated syndromes that could also benefit from re-evaluation in the light of these new genetic findings.
